# Spectrum Sensing Method Based on Residual Dense Network and Attention

**DOI:** 10.3390/s23187791

**Published:** 2023-09-11

**Authors:** Anyi Wang, Qifeng Meng, Mingbo Wang

**Affiliations:** School of Communication and Information Engineering, Xi’an University of Science and Technology, Xi’an 710054, China; wanganyi@xust.edu.cn (A.W.); 21207040026@stu.xust.edu.cn (Q.M.)

**Keywords:** cooperative spectrum sensing, residual dense network, attention mechanism

## Abstract

To address the problems of gradient vanishing and limited feature extraction capability of traditional CNN spectrum sensing methods in deep network structures and to effectively avoid network degradation issues under deep network structures, this paper proposes a collaborative spectrum sensing method based on Residual Dense Network and attention mechanisms. This method involves stacking and normalizing the time-domain information of the signal, constructing a two-dimensional matrix, and mapping it to a grayscale image. The grayscale images are divided into training and testing sets, and the training set is used to train the neural network to extract deep features. Finally, the test set is fed into the well-trained neural network for spectrum sensing. Experimental results show that, under low signal-to-noise ratios, the proposed method demonstrates superior spectral sensing performance compared to traditional collaborative spectrum sensing methods.

## 1. Introduction

In the evolving global economy and advancements in information technology, the demand for spectrum resources within the 5G frequency band is consistently rising. However, the limited availability of these spectrum resources hampers the high-speed, large-capacity growth of 5G networks [1] and the swift proliferation of the Internet of Things (IoT) [2]. To optimize the use of these resources, cognitive radio (CR) has been introduced as an efficacious solution [3]. This technology permits secondary users (SU) to access idle frequency bands without disrupting the operations of primary users (PU), thus significantly enhancing the utilization of spectrum resources.

Spectrum sensing is a fundamental requirement for enabling dynamic spectrum access in cognitive radio [4,5]. This technology assists secondary users in swiftly and accurately identifying and utilizing idle spectrum, thereby enhancing their probability of spectrum access [6]. The primary categories of spectrum sensing are single-user spectrum sensing and collaborative spectrum sensing. Reference [7] primarily investigates a spectrum sensing method based on the Kernelized Energy Detector (KED). Compared to the traditional Energy Detector (ED), this method offers greater accuracy, especially in non-Gaussian noise environments. Reference [8] focuses on improving the ED in a Laplacian noise setting and introduces a new test statistic to enhance detection performance. Traditional single-user spectrum sensing is susceptible to challenges such as a low signal-to-noise ratio (SNR), pronounced shadow fading, and the presence of hidden terminals. While collaborative spectrum sensing can mitigate these issues to some degree, it often grapples with determining an appropriate threshold value in real-world settings. Recent studies have explored spectrum sensing through machine-learning techniques. Unlike traditional methods, these algorithms eliminate the need for intricate threshold derivations and can efficiently classify and recognize signals after classifier training. For instance, literature [9] employed a support vector machine (SVM) trained on signal energy vector features. However, this approach exhibits subpar detection performance in low SNR conditions, and training the SVM is notably time-consuming. Deep learning [10,11] has been extensively applied in image processing [12,13], speech recognition [14], natural language processing [15], and radio signal classification [16,17,18]. Additionally, deep learning has also found applications in the medical field [19,20]. Convolutional neural networks (CNNs) are effective tools for extracting target features and offer superior performance in 2D image feature extraction compared to other machine learning algorithms. In the study presented in [21], signal recognition is achieved by normalizing the cyclic spectrum of OFDM (Orthogonal Frequency Division Multiplexing) signals to grayscale, creating a cyclic autocorrelation grayscale image, and subsequently training a CNN. However, this approach is limited to single-user spectrum sensing. Another study [22] introduces a spectrum sensing technique that utilizes the signal covariance matrix. This method involves converting the covariance matrix into a grayscale image and feeding it into the CNN model for spectrum sensing. Nevertheless, the capacity of the CNN model to extract features is constrained. Additionally, as the depth of the model increases, CNNs may encounter the gradient vanishing issue, leading to suboptimal accuracy in spectrum sensing.

The Residual Neural Network (ResNet) [23] effectively addresses the gradient vanishing issue and mitigates overfitting through the incorporation of shortcut connections within the CNN architecture. This allows for the expansion of network layers. Nevertheless, ResNet does not fully harness the information from the output of each layer within the residual block, nor does it acknowledge the interrelation between distinct convolutional layers. By seamlessly integrating dense connections into ResNet and ensuring that all neurons from one layer connect with the subsequent layer, we enhance the interchange of feature extraction information between any pair of convolutional layers. This led to the introduction of the Residual Dense Network (RDN). However, with increasing network depth, feature degradation becomes a concern. To address this, we embed the Convolutional Block Attention Module (CBAM) within the RDN. CBAM prioritizes computational resources for salient features while diminishing attention to less crucial ones, thereby optimizing image feature extraction.

In conclusion, this study introduces an OFDM (Orthogonal Frequency Division Multiplexing) cooperative spectrum sensing technique utilizing Residual Dense Networks and the Convolutional Block Attention Module (RDN–CBAM). This approach involves converting signal time-domain data into a two-dimensional matrix, which is subsequently normalized and transformed into a grayscale image. This image is partitioned into training and test sets. The training set facilitates the neural network’s learning to extract intricate features from the grayscale image. Ultimately, the test set is fed into the trained neural network spectrum sensing model, recasting the spectrum sensing challenge as an image classification task.

## 2. System Model

Spectrum sensing is the process through which secondary users perceive whether primary users are present in a specific channel. Thus, spectrum sensing can be transformed into a classic binary hypothesis testing problem. It respectively corresponds to the non-existence and existence of the primary user, so this problem can be expressed as follows:

(1) $$\{\begin{array}{c} H_{1}:y\left(n\right)=x\left(n\right)+w\left(n\right)\\ H_{0}:y\left(n\right)=w\left(n\right) \end{array},$$

In the formula, $H_{1}$ and $H_{0}$, respectively, represent the hypotheses for spectrum occupancy and idleness. y(n) represents the received signal, x(n) represents the transmitted signal from the primary user, and w(n) is the Gaussian noise with a mean of 0 and variance of σ^2^. $P_{d}$ and $P_{fa}$, respectively, represent the probabilities of detection and false alarm. This paper will use these two metrics to evaluate the performance of the spectrum sensing model. $P_{d}$ and $P_{fa}$ can be expressed as:

(2) $$P_{d}=P\left\{H_{1}|H_{1}\right\},$$

(3) $$P_{fa}=P\left\{H_{1}|H_{0}\right\},$$

## 3. Spectrum Sensing Algorithm Based on RDN–CBAM for OFDM

**Figure 1:**
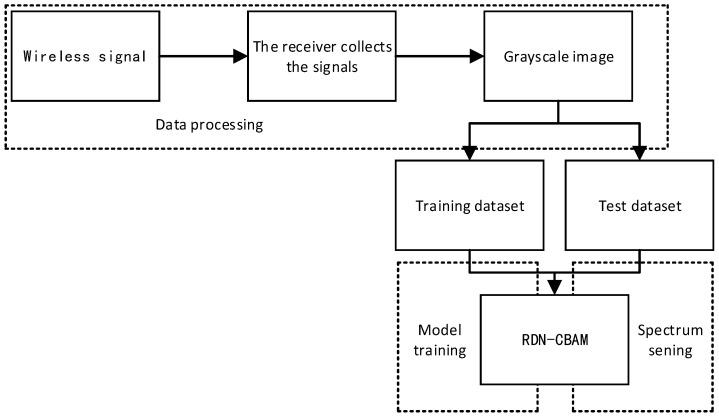
Framework of the spectrum sensing system.

The process of the spectrum sensing method in this paper consists of three parts: data processing, model training, and spectrum sensing, as shown in Figure 1.

### 3.1. Data Processing

Due to the differences in the overall distribution of sampling point values for unauthorized users under $H_{1}$ and $H_{0}$ conditions, the brightness presented by the grayscale image is also different. Utilizing this difference, the received signals are sampled, and the matrix after sampling is normalized and processed into grayscale images. The received signals are represented as:

(4) y(n)=I(n)+jQ(n),n=1,2,…,N

In the equation, I(n) and Q(n), respectively, represent the *I*-path and *Q*-path signals received by the SU. The *Q*-path signal is intentionally modulated during transmission; thus, this paper only uses the *I*-path signal to construct the dataset. The normalized *I*-path signal, denoted as $\bar{I}\left(n\right)$, is obtained as follows:

(5) $$\bar{I}\left(n\right)=\frac{I\left(n\right)-I_{min}}{I_{max}-I_{min}},$$

The matrix $X_{\bar{I}}$ is constructed by sequentially stacking $\bar{I}\left(n\right)$ signals, and its expression is given by:

(6) $$X_{\bar{I}}=\left[\begin{array}{ccc} \bar{I}\left(1\right) & \bar{I}\left(2\right) & \begin{array}{cc} \cdots & \bar{I}\left(w\right) \end{array}\\ \bar{I}\left(w+1\right) & \bar{I}\left(w+2\right) & \begin{array}{cc} \cdots & \bar{I}\left(2w\right) \end{array}\\ \begin{array}{c} \vdots \\ \bar{I}\left(k-1\right)\left(w+1\right) \end{array} & \begin{array}{c} \vdots \\ \bar{I}\left(k-2\right)\left(w+2\right) \end{array} & \begin{array}{cc} \begin{array}{c} \ddots \\ \cdots \end{array} & \begin{array}{c} \vdots \\ \bar{I}\left(kw\right) \end{array} \end{array} \end{array}\right],$$

**Figure 2:**
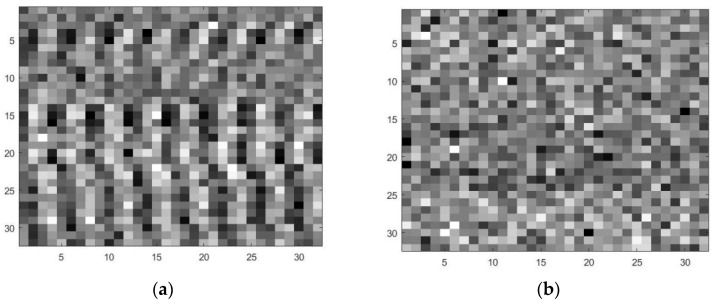
(**a**) $H_{1}$ grayscale image; (**b**) $H_{0}$ grayscale image.

Here, *w* and *k* represent the number of rows and columns in the matrix, respectively. The matrix $X_{\bar{I}M}$ is constructed by stacking $X_{\bar{I}}$, and the formula is given by:

(7) $$X_{\bar{I}M}=\left[\begin{array}{c} \begin{array}{c} X_{I1}\\ X_{I2} \end{array}\\ \begin{array}{c} \vdots \\ X_{IM} \end{array} \end{array}\right],$$

where *M* represents the number of secondary users. By mapping the values in matrix $X_{\bar{I}M}$ to the grayscale values of the grayscale image, it is transformed into a grayscale image, as shown in Figure 2.

### 3.2. Residual Connection

Residual connections are utilized to address the issues of gradient vanishing and network degradation in deep neural networks. Their introduction aimed to overcome the difficulties in training deep networks, allowing information to bypass several layers directly within the network, thereby retaining and transmitting more useful information.

In traditional CNNs, information is passed through each level layer by layer, going through a series of transformations and non-linear activation functions to eventually produce an output. However, when the network is exceptionally deep, information may gradually vanish or get lost after passing through numerous transformations, leading to a decrease in network performance. This is because the gradient may become very small during backpropagation, making it challenging to update the weights. Furthermore, increasing the number of network layers can potentially make the training process unstable.

The introduction of residual connections can solve this problem. Specifically, they add a direct connection between the input and output at each level. This connection allows information to pass directly through the network, bypassing the transformation process of some layers. By adding the input directly to the output, the network can retain the original information and adjust and correct it in subsequent layers.

Mathematically, residual connections can be represented as:

(8) y(n)=f(x)+x,

where x is the input, f(x) represents the residual mapping of the feature map, and y(n) is the output. By adding such residual connections, it can be ensured that even in the deep parts of the network, the gradient can still propagate directly to the early layers, thereby avoiding the vanishing gradient problem.

### 3.3. Dense Connections

Densely connected structures are utilized to enhance feature propagation and information flow in neural networks. Compared to the traditional layer-by-layer connections of conventional CNNs, densely connected structures establish direct connections with all previous layers at every level, enabling global information exchange and feature reuse.

In traditional CNNs, the input of each layer is only connected to the output of the previous layer, and information needs to be passed on layer by layer to reach subsequent levels. This connection method may lead to the gradual reduction or loss of information during transmission, limiting the network’s learning and representation capabilities.

This issue can be addressed with dense connections. In the output of each layer, the outputs of all preceding layers are connected. This implies that the input of each layer consists of the outputs from all previous layers, forming a densely connected pattern. This type of connection allows information to freely flow and propagate within the network, permitting features from lower layers to directly participate in the decision-making of higher layers.

Dense connections can be represented as:

(9) $$y=H\left(\left[x_{1},x_{2},x_{3},\ldots ,x_{n-1},x_{n}\right]\right),$$

In this equation, x represents the input, y represents the output of the layer, H(·) denotes the mapping of the feature map, and [·] square brackets signify the concatenation of multiple inputs.

Through dense connections, each layer can directly access the information from all previous layers, thereby enabling global interaction and sharing of features. This global information transmission aids the network in better learning feature representations, thereby enhancing the network’s expressive power and learning capability.

### 3.4. Residual Dense Network

RDN is a deep convolutional neural network used for image super-resolution reconstruction, adopting the structure of residual connections and dense connections.

In traditional convolutional neural networks, the input of each layer is the output of the previous layer. However, this might lead to issues such as gradient vanishing and gradient explosion. To address this issue, residual connections are introduced into RDN. Residual connections add the input to the output within each residual block, allowing the network within each block to learn the difference between the input and the output. This makes the training more stable and enables a deeper network depth.

On the other hand, dense connections link the input and output of each block, allowing the network to utilize all information from previous layers for training. This enhances the network’s feature extraction ability and alleviates the gradient vanishing problem. Therefore, RDN can effectively increase network depth and achieve better performance.

**Figure 3:**
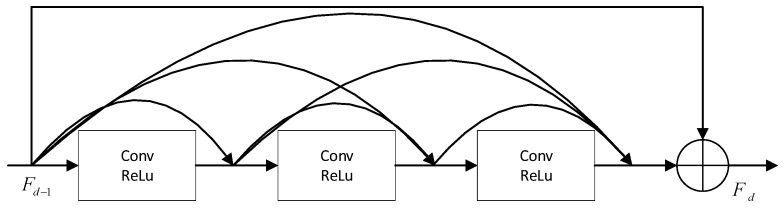
RDB structure diagram.

The basic units of the RDN, the Residual Dense Block (RDB), are composed of the structure of residual connections and dense connections, which contains a composite function with batch Normalization (BN), ReLU, Conv, and other operations. The d-th RDB is shown in Figure 3.

### 3.5. Convolutional Block Attention Module (CBAM)

CBAM is a module used to enhance the performance of convolutional neural networks. The CBAM module combines two attention mechanisms, the channel attention mechanism, and spatial attention mechanism, aiming to capture the inter-channel relationships and spatial correlations in the input feature map, thereby improving the expressiveness and generalization ability of the model.

The basic structure of the CBAM module includes two sub-modules: channel attention module and spatial attention module. The channel attention module is used to capture the correlations among different channels in the input feature map. It performs average pooling and max pooling along the spatial dimension of the feature map, then concatenates their results into a fully connected layer to generate a channel attention vector. This vector is used to weight each channel, enhancing the important channel information while suppressing the useless channel information. The formula for the channel attention mechanism is as follows:

(10) $$M_{c}\left(F\right)=\sigma \{MLP[avgpool\left(F\right)]+MLP\left[maxpool\left\{F\right\}\right]\}=\sigma \left\{W_{1}\left[W_{0}\left(F_{avg}^{c}\right)+\;W_{1}\left[F_{max}^{c}\right]\right]\right\},$$

where $F_{avg}^{c}$ and $F_{max}^{c}$, respectively, represent the feature maps outputted by the average pooling operation and the maximum pooling operation of the channel attention mechanism. $W_{1}$ and $W_{0}$ denote the weights of the multi-layer perceptron, while σ signifies the sigmoid function. $M_{c}\left(F\right)$ represents the output of the channel attention mechanism.

The Spatial Attention Module is utilized to capture the correlation between different spatial positions within the input feature maps. It conducts both average pooling and maximum pooling along the channel dimension of the feature maps, then concatenates their results into a fully connected layer, generating a spatial attention vector. This vector is applied to weight each spatial position, enhancing relevant position information and suppressing irrelevant position information. The formula for the spatial attention mechanism is:

(11) $$M_{s}\left(F\right)=\sigma \left(f^{7\times 7}\left\{\left[avgpool\left(F\right);maxpool\left\{F\right\}\right]\right\}\right)=\sigma \left\{f^{7\times 7}\left[\left(F_{avg}^{s};F_{max}^{s}\right)\right]\right\}$$

where $F_{avg}^{s}$ and $F_{max}^{s}$ represent the feature maps outputted by the average pooling operation and the maximum pooling operation of the spatial attention mechanism, respectively. The symbol $f^{7\times 7}$ denotes the size of the convolution kernel, while $M_{s}\left(F\right)$ stands for the output of the spatial attention mechanism.

**Figure 4:**
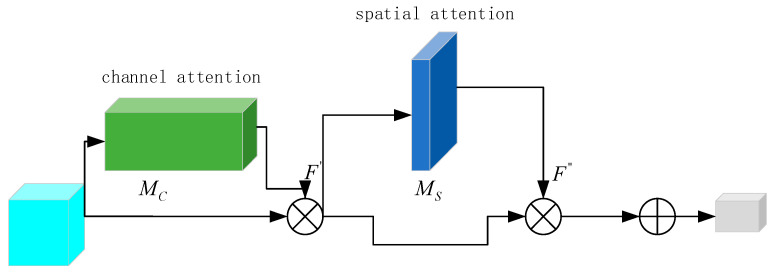
CBAM structure diagram.

The CBAM module combines channel attention and spatial attention to create a comprehensive attention map. This takes into account both the inter-channel relationships and spatial correlations of the input feature maps, thereby enhancing the model’s expressiveness and generalization ability. The structure of the CBAM is shown in Figure 4.

**Figure 5:**
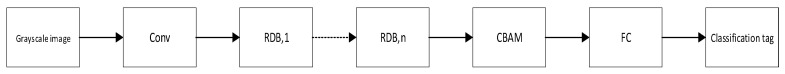
CBAM structure diagram ResDenNet-CBAM spectrum sensing model.

The proposed deep RDN–CBAM spectrum sensing model in this paper consists of an input layer, a shallow feature extraction layer (Conv), multiple RDB modules, CBAM, FC (Fully Connected) layers, and classification labels. The structure of the ResDenNet-CBAM spectrum sensing model is shown in Figure 5.

In this paper, the internal convolution kernel size of the RDB is set to 1 × 1, and the Conv has a kernel size of 3 × 3 with 8 kernels in each layer.

In the RDN–CBAM spectrum sensing algorithm, we select n pairs of data $\left\{\left(x^{\left(1\right)},y^{\left(1\right)}\right),\ldots ,\left(x^{\left(n\right)},y^{\left(n\right)}\right)\right\}$ as the training set and *m* pairs of data $\left\{\left(x^{\left(n+1\right)},y^{\left(n+1\right)}\right),\ldots ,\left(x^{\left(n+m\right)},y^{\left(n+m\right)}\right)\right\}$ as the testing set, where $x^{\left(\cdot \right)}$ represents the grayscale image of the received signal and $y^{\left(\cdot \right)}$ represents the label value of the received signal. The input-output mapping relationship in this algorithm is given by:

(12) $$f_{w,b}\left(x^{\left(n\right)}\right)={\hat{y}}^{\left(n\right)},$$

where *w* and *b* are the trained weights and biases in the network, ${\hat{y}}^{\left(n\right)}$ representing the output mapping of $x^{\left(n\right)}$ after passing through the network.

The loss function for training RDN–CBAM is represented as:

(13) $$Loss=-\sum_{i=1}^{2}y^{\left(n\right)}\left(i\right)log\left({\hat{y}}^{\left(n\right)}\left(i\right)\right),$$

The proposed RDN–CBAM spectrum sensing algorithm is shown in Algorithm 1.
**Algorithm 1.** Spectrum Sensing Algorithm of RDN–CBAM.1: Input: training and test set sample data  Output: detection probability and false alarm probability  2: Input the training set samples $\left\{\left(x^{\left(1\right)},y^{\left(1\right)}\right),\ldots ,\left(x^{\left(n\right)},y^{\left(n\right)}\right)\right\}$.  3: The predicted label ${\hat{y}}^{\left(n\right)}$ is updated according to Equation (10); Backpropagation is performed to update the loss based on Equation (11) until convergence.  4: Apply the trained RDN–CBAM model to the test data $\left\{\left(x^{\left(n+1\right)},y^{\left(n+1\right)}\right),\ldots ,\left(x^{\left(n+m\right)},y^{\left(n+m\right)}\right)\right\}$. Calculate the correct number $r_{PU}$ of PU identification and the correct number $r_{SU}$ of noise sample identification.5: Finally, the detection probability $P_{d}=\frac{r_{PU}}{n}$ and false alarm probability $P_{fa}=\frac{r_{SU}}{n}$ are calculated.

## 4. Experimental Analysis

### 4.1. Experimental Conditions

To validate the performance of the RDN–CBAM spectral perception algorithm, we conducted the following simulation experiments and specified the parameter settings used in the experiments. In the simulation experiments, the CPU and GPU used were Intel Core i7 and NVIDIA GeForce 3060, respectively. We used Matlab 2020a to generate OFDM signals and selected OFDM signals with signal-to-noise ratios ranging from −20 to 0 dB at 2 dB intervals as experimental data, setting the sampling point *N* = 256 and the number of cooperative users M as 20. The parameters for the OFDM signal model are shown in Table 1. The loss function for model training was a cross-entropy loss function. During training, the Adam optimization algorithm [24] was used for optimization, with batch size set to 16 and learning rate set to 0.001.

### 4.2. Experiment 1: Impact of Network Depth on Model Performance

**Figure 6:**
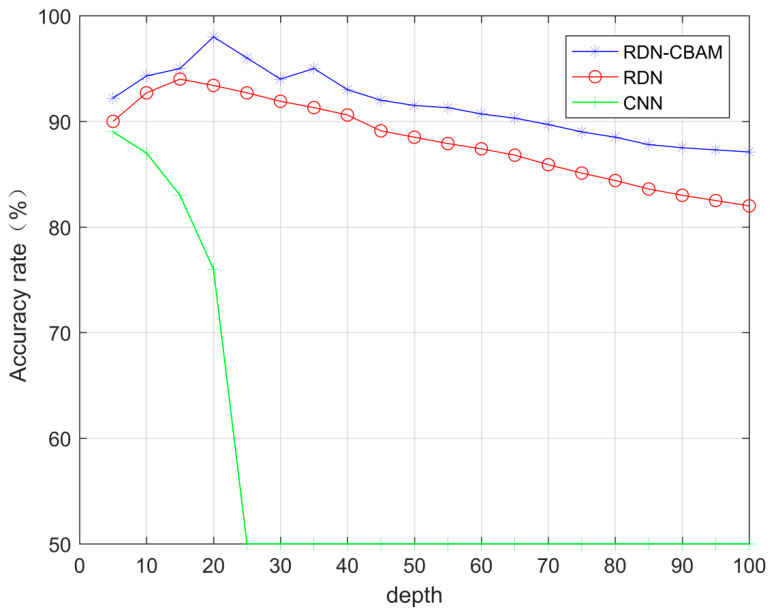
RDN–CBAM, RDN, and CNN accuracy change with the number of network layers.

In this section of the experiment, the SNR range of the OFDM signals was −20 to 0 dB, with intervals of 2 dB. An equal amount of OFDM signal data and noise data were collected under each SNR condition. For each SNR, 100 sets of data were selected, forming a total of 1100 sets of signal data and 1100 sets of noise data. To train the model, we randomly selected 990 sets of signal data and 990 sets of noise data as the training set and chose 110 sets of signal data and 110 sets of noise data as the test set. In the experiment, the convolutional layer numbers of RDN–CBAM, RDN, and CNN were the same. Figure 6 displays the change in classification accuracy of RDN–CBAM, RDN, and CNN with the increase in network layers under the spectrum sensing method.

As shown in Figure 6, when the number of network layers is less than 20, the accuracy of RDN–CBAM continuously increases, reaching its peak at 20 layers. RDN achieves its highest accuracy at 16 layers. As the number of layers increases, the accuracy begins to decline. The accuracy of CNN continues to drop to 50%, while the accuracy of RDN–CBAM is consistently higher than both RDN and CNN. As the number of network layers gradually increases, the accuracy of RDN–CBAM slowly drops to around 87%, and RDNs accuracy decreases to about 83%. Meanwhile, the accuracy of CNN remains steady at 50%. The increase in the number of network layers leads to the vanishing gradient problem in CNN, resulting in a decrease in the model’s accuracy. However, the deep RDN–CBAM can effectively extract more image features, ensuring its accuracy is consistently higher than that of RDN. Having excessively deep network layers can also cause a decline in the performance of RDN–CBAM, as overfitting may occur during training with very deep RDN–CBAM layers. Yet, the performance degradation is much slower than in RDN. This is because, as the depth of the network increases, RDN is prone to feature degradation issues. In conclusion, since RDN–CBAM achieves the highest accuracy when the network has 20 layers, the RDN–CBAM model used in this study has been chosen to have 20 layers.

**Figure 7:**
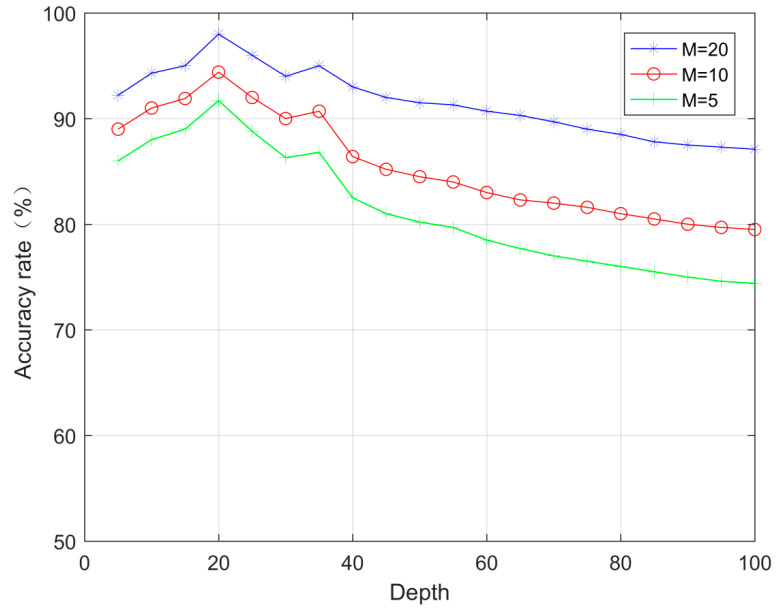
Influence of the number of secondary users on the accuracy of RDN–CBAM spectrum sensing algorithm with different layers.

As shown in Figure 7, when the number of network layers is constant, the accuracy of the RDN–CBAM spectrum sensing algorithm increases with the rise in the number of secondary users. This is because as the number of these users increases, the features of the signal mapped to the grayscale image also increase, thus enhancing the accuracy of the RDN–CBAM spectrum sensing algorithm. This study selects the spectrum sensing algorithm with 20 secondary users.

### 4.3. Experiment 2: Influence of Residual Structure on Model Gradient

**Figure 8:**
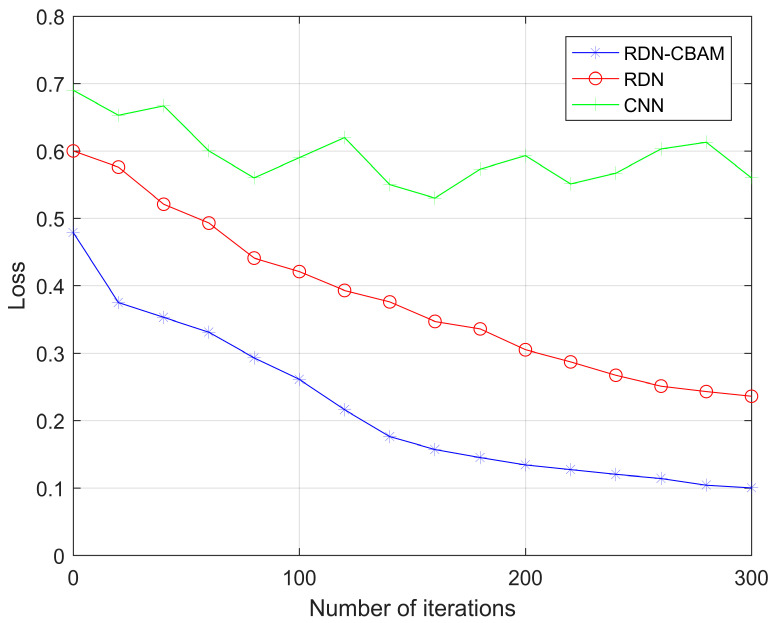
RDN–CBAM, RDN, and CNN loss change with the number of iterations.

**Figure 9:**
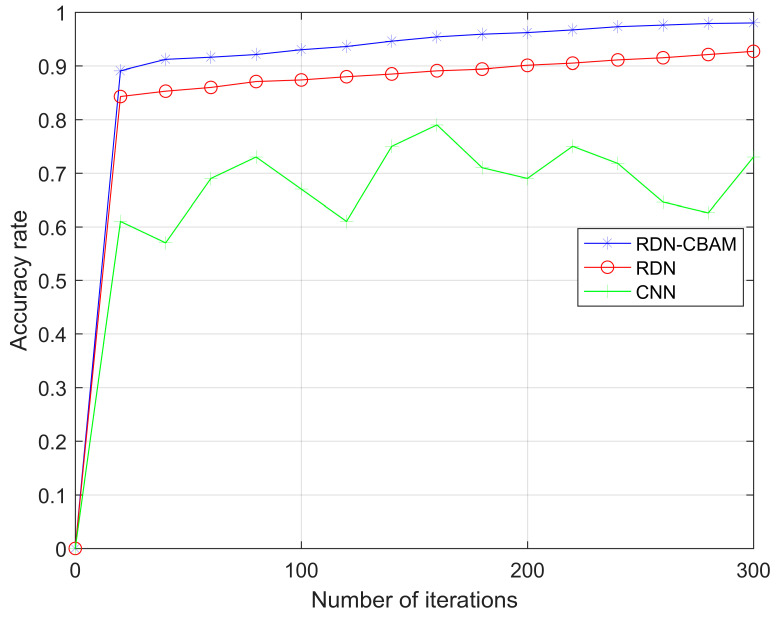
The change of accuracy rate of RDN–CBAM, RDN, and CNN with the number of iterations.

In this experiment, we selected 20 layers as the network depth for RDN–CBAM, RDN, and CNN, using the same dataset as Experiment 1. As shown in Figure 8 and Figure 9, the loss for RDN–CBAM eventually stabilizes at around 0.1, which is notably lower than that for RDN (around 0.25) and CNN (around 0.56). Moreover, the accuracy rate for RDN–CBAM remains steady at around 0.98, surpassing RDN (around 0.92) and CNN (around 0.53). RDN–CBAM, equipped with a residual structure and convolutional attention, can prevent gradient vanishing and enhance feature extraction capabilities, thereby increasing accuracy and reducing loss. This strongly suggests that incorporating a residual structure into CNN can effectively address the gradient vanishing problem and enhance network performance.

### 4.4. Experiment 3: Comparison of Sensing Efficiency among RDN–CBAM, CNN, and SVM Spectral Sensing Methods

In the experiment, both RDN–CBAM and CNN used networks of 5 layers (represented as RDN–CBAM_5L and CNN_5L) and 20 layers (represented as RDN–CBAM_20L and CNN_20L), with all other parameters being consistent. The training and validation sets remained the same as in Experiment 1.

Table 2 compares the performance of several different spectral sensing methods in terms of training time, testing time, and other aspects. From Table 2, it can be observed that compared to CNN_5L, the training time of RDN–CBAM_5L was reduced by 3.2 s, and the testing time was reduced by 1.02 s. Compared to CNN_20L, RDN–CBAM_20L had its training time reduced by 4.56 s and testing time reduced by 1.78 s. The reason behind this is that the residual connections accelerated the convergence speed of RDN–CBAM, effectively reducing both training and testing times. Compared to SVM, although the training time for RDN–CBAM_20L increased by 18.18 s, its testing time was reduced by 6.39 s. This is because RDN–CBAM_20L has more training parameters than SVM, leading to a longer training period. During the testing phase, RDN–CBAM can directly recognize and classify received signals, whereas SVM requires retraining of its classifier before it can identify received signals. Therefore, the testing time for RDN–CBAM is faster. In a real CR environment, since the best-performing spectral sensing classifier has been pretrained, it can be directly used for spectral sensing. Hence, testing time becomes particularly important in practical sensing, demonstrating that the method proposed in this paper is both efficient and feasible.

From a complexity perspective, let n denote the number of training samples and m represent the number of unauthorized users. The SVM algorithm needs to compute matrix eigenvalues and carries out classifications with a complexity of $O\left(n^{3}\right)$, resulting in an overall complexity of $O\left(nm^{3}+n^{3}\right)$. The complexity of the CNN spectrum sensing algorithm is $O\left(n\sum_{L=1}^{L}F_{l}^{2}K_{l}^{2}Q_{l}Q_{l-1}\right)$, where *L*, $F_{l}$, $K_{l}$*,* and $Q_{l}$, respectively, indicate the number of network layers, the edge length of the feature map output from the $l^{th}$ Conv, the side length of the convolution kernel, and the number of output channels.

The sole difference in complexity between the RDN–CBAM spectrum sensing algorithm and the CNN algorithm lies in the different number of layers l. The RDN–CBAM spectrum sensing algorithm can skip one or more Conv layers through shortcut connections. This allows the l in $O\left(n\sum_{L=1}^{L}F_{l}^{2}K_{l}^{2}Q_{l}Q_{l-1}\right)$ to “jump” in selecting the number of layers, instead of having the complexity l accumulate from the first layer all the way to the $l^{th}$ layer as in the CNN algorithm. As a result, RDN reduces the parameters required to train convolution layers, thus decreasing the algorithm’s complexity.

Given that CBAM is a lightweight and versatile module, the overhead of this module can be neglected, allowing its seamless integration into any CNN architecture. Hence, the overall complexity of the RDN–CBAM spectrum sensing algorithm can be reduced.

### 4.5. Experiment 4: Comparison of Spectrum Sensing Performance among Different Models

**Figure 10:**
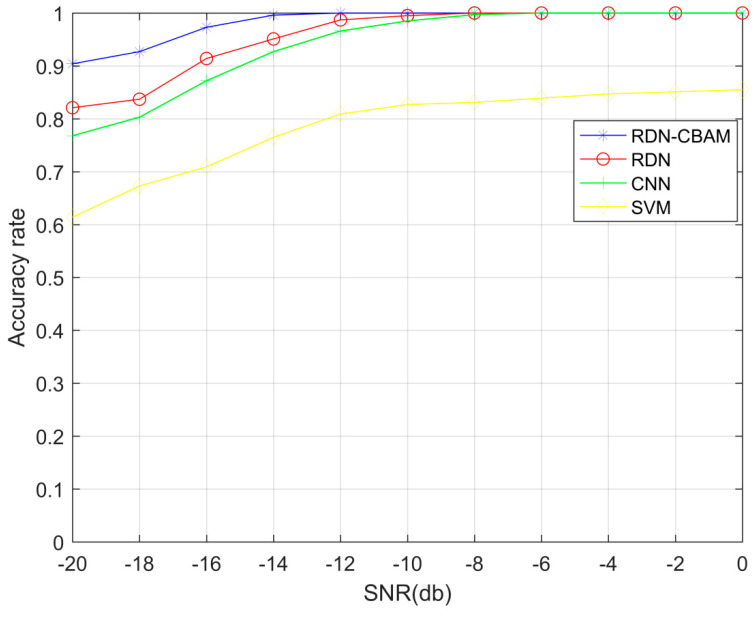
Detection probabilities of RDN–CBAM, RDN, CNN, and SVM under different SNR.

In our experiment, we compared the detection probabilities of RDN–CBAM, RDN, CNN, and SVM spectral sensing methods under SNR values ranging from −20 dB to 0 dB. The number of layers chosen for the RDN–CBAM, RDN, and CNN spectral sensing models were 20 layers, 16 layers, and 5 layers, respectively, using the same dataset as Experiment 1. The experimental results, as shown in Figure 10, reveal that when the SNR is lower than −10 dB, the detection probability of the RDN–CBAM spectral sensing method consistently surpasses the other methods, including RDN, CNN, and SVM. This is because the convolutional attention module of RDN–CBAM can extract more useful features and suppress irrelevant ones, thereby effectively enhancing the model’s detection capabilities.

We conducted multiple experiments to comprehensively validate the effectiveness of the RDN–CBAM spectrum sensing method and recorded the $P_{d}$ and $P_{fa}$ values in each experiment compared to the traditional CNN, RDN, and SVM spectrum sensing methods, obtaining the ROC (Receiver Operating Characteristic) curve. In these experiments, we used the same dataset as in Experiment 1, taking SNR = −16 dB as an example.

**Figure 11:**
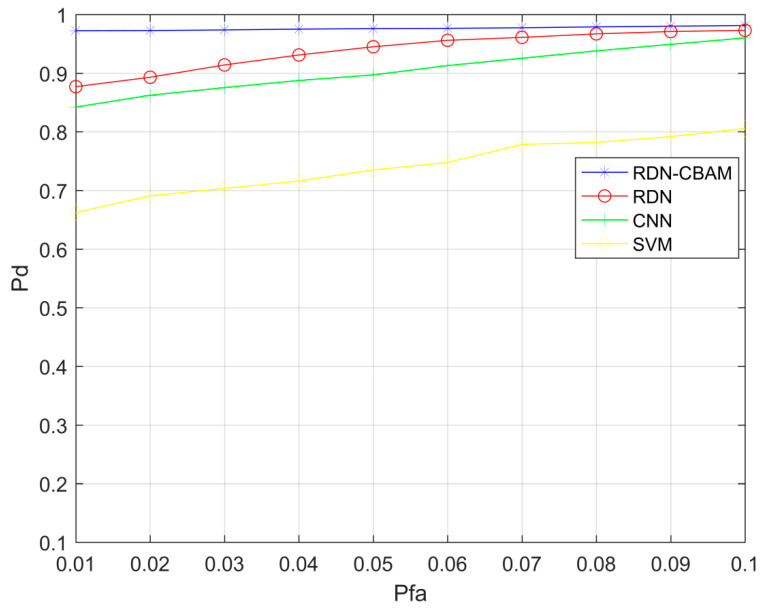
ROC curves of RDN–CBAM, RDN, CNN, and SVM spectrum sensing methods.

Figure 11 displays the experimental results. We can observe that the RDN–CBAM spectrum sensing method performed exceptionally well in all experiments. At a false alarm probability $P_{fa}$ = 0.05, the detection probabilities for RDN–CBAM, RDN, CNN, and SVM methods were 0.97, 0.91, 0.87, and 0.71, respectively. The ROC curve for RDN–CBAM is notably higher than those for the CNN, RDN, and SVM spectrum sensing methods. This indicates that the RDN–CBAM spectrum sensing method exhibits strong performance in processing low signal-to-noise ratio signals and can significantly enhance the accuracy and reliability of spectrum sensing.

## 5. Conclusions

To improve the feature extraction capability of traditional CNN spectral perception methods and to avoid the problem of gradient vanishing in deep network structures, as well as to address the feature degradation issue when increasing the layers of the RDN network, we introduce an RDN–CBAM spectral perception approach. This method employs residual dense blocks and convolutional attention mechanisms to thoroughly extract deep features of grayscale images. Furthermore, it uses a deep network to train the spectral perception model to enhance the classification and recognition accuracy of grayscale images, thereby improving spectral perception performance. Experimental results show that, compared to spectral perception methods like CNN, RDN, and SVM, our proposed RDN–CBAM spectral perception approach has a higher detection probability under equal false alarm rates and a lower false alarm rate under equal detection probabilities. Moreover, there is no issue of network degradation in deep network structures.

This paper aims to address the shortcomings of traditional CNN models, namely the vanishing gradient problem and weak feature extraction. Future work will focus on testing the performance of the proposed model on different modulated signals and comparing it with more advanced models.

## Figures and Tables

**Table 1:** OFDM parameters.

Simulation Parameters	Parameter Value
Number of OFDM symbols	20
Number of subcarriers	128
Symbol rate	12.5 KHz
Cyclic prefix ratio	0.25
Carrier frequency	10 MHz
Sampling frequency	40 MHz
Chip frequency	0.5 MHz
Smoothing points	16

**Table 2:** $P_{d}$, $P_{fa}$, Training Time, and Sensing Time of Several Algorithms.

Algorithm	Training Time/s	Sensing Time/s
RDN–CBAM_5L	21.43	2.23
CNN_5L	24.92	2.74
RDN–CBAM_20L	32.75	3.93
CNN_20L	37.31	5.71
SVM	14.57	10.32

## Data Availability

Not applicable.
